# eDNA for detection of five highly invasive molluscs. A case study in urban rivers from the Iberian Peninsula

**DOI:** 10.1371/journal.pone.0188126

**Published:** 2017-11-15

**Authors:** Laura Clusa, Laura Miralles, Ana Basanta, Carmelo Escot, Eva García-Vázquez

**Affiliations:** 1 Department of Functional Biology, University of Oviedo, Oviedo, Asturias, Spain; 2 Metropolitan Water Supply and Sanitation Company of Sevilla, EMASESA., Sevilla, Spain; University of Hyogo, JAPAN

## Abstract

Biological invasions are an important threat to biodiversity especially in aquatic ecosystems, and their frequency is generally higher near urban areas. Potentially invasive non-indigenous molluscs were deliberately introduced into European waters for food (*Corbicula fluminea*) and biocontrol (*Melanoides tuberculata*), and unintentionally introduced by ballast water (*Mytilopsis leucophaeata*, *Corbicula fluminea)*, stock contamination (*Sinanodonta woodiana)*, accidental escapes from aquaculture (*Sinanodonta woodiana*), aquarium trade releases (*Melanoides tuberculata*) and even attached to aquatic birds (*Corbicula fluminea*). Three rivers from the Iberian Peninsula were monitored near the three most populated inland cities to evaluate the presence of these invasive molluscs through PCR amplification using taxon-specific primers from eDNA. New primers were designed within 16S rRNA and cytochrome oxidase subunit I genes, tested *in silico* from BLAST methodology and experimentally *in vitro* before application in the field. *C*. *fluminea* was found in Ebro River (near Zaragoza); *M*. *leucophaeata* in Guadalquivir River (near Sevilla). *M*. *tuberculata* and *S*. *woodiana* were found from enclosed areas (lake and reservoir respectively) upstream, respectively, Zaragoza and Madrid. The new tools are ready to be used in other regions where these species are also invasive.

## Introduction

Biological invasions are one of the most important threats to biodiversity. Particularly in aquatic ecosystems the number of invasive species has increased in the last decades, due to globalization and closely related to human activities [1, 2]. Human-mediated transport together with global warming could promote the rapid and uncontrolled dispersion of invasive freshwater species [3]. An example is the rapid spread of *Mytilopsis leucophaeata* (native to the Gulf of Mexico) and the zebra mussel *Dreissena polymorpha* (native to the Caspian Sea) in the Baltic Sea [4].

The ways of introduction of aquatic species are numerous. Invertebrates are deliberately introduced for food and biocontrol, and may be also unintentionally released from accidental aquaculture escapes, ballast water, water connections, hitchhikers, stock contamination, pet and aquarium trade [5, 6]. For example, *M*. *leucophaeata* was introduced in Baltic Sea as well as in Guadalquivir River in Spain by ballast water transport [7, 8]. Multiple introductions of the same species have also been reported. *Melanoides tuberculata*, native from eastern Africa and the Middle East was deliberately introduced in 1980 for snail control in the Caribbean [9], and also inadvertently from aquarium trade [10]. *Sinanodonta woodiana* whose native range is Eastern Asia, was introduced in Tuscany (Italy) for production of artificial pearls [11], but in Poland it arrived probably with fish consignments as a parasite [12], since it has an obligatory parasitic stage [13]. *Corbicula fluminea*, also native to Asia, was transported inadvertently in ballast water to Brazil [14], where it was also introduced from aquarium releases and attached to feet or feathers of aquatic birds, and deliberately released as a food resource and fish bait [15].

The effects caused by the molluscs cited above are enormous and mainly derived from their high reproductive rates and their environmental tolerance. They can alter the suspended particles and sediments, introduce new parasites and diseases and compete with native species [16]. The parthenogenetic *M*. *tuberculata* is a threat for the Italian endemic *Melanopsis etrusca* due to its high population density [11]. Moreover *M*.*tuberculata* hosts a trematode parasite that infests local fish [17]. *C*. *fluminea* reduces the local phytoplankton community due to high filtration rates, altering the nutrients cycling [18], as it happened in the Potomac River in Maryland, USA [19]. In addition, this species tolerates higher concentrations of mercury than native molluscs, surviving better in polluted areas [20]. *S*. *woodiana* became the dominant species in Poland and dispersing quickly to the rest of Europe [21], partly due to its high ability to parasitize native fish species outcompeting native molluscs [22]. *M*. *leucophaeata* is even able to survive in the cooling tanks of nuclear plants and spread from there [23]. Its ability to tolerate high temperatures and chlorine concentrations makes *M*. *leucophaeata* a huge biofouling problem once established [24, 25] as it happened with *D*. *polymorpha* [26]. Even the empty shells of these molluscs can cause serious damage to the ecosystem. In the Danube empty shells of *C*. *fluminea* and *S*. *woodiana* accumulated, sheltering amphipods and isopods and attracting predator populations [27]. The examples above illustrate the impact of these invasive molluscs in European and other freshwaters. The human population density is one of the best predictors of biological invasions [28–31]. As other invasive species, exotic molluscs tend to accumulate near big cities and ports, in highly anthropogenic areas where the invasion vectors accumulate [32]: ballast water, hull fouling, aquarium wastes, pet releases e.g [33, 34]. Thus water bodies nearby the cities should be logically main monitoring targets, in order to control potential entries of new undesired species; especially when those species, as the molluscs cited above, have been already reported as biological invasions in the same and/or other regions.

Sousa *et al*. [16] suggested using novel tools such as environmental DNA (eDNA) for the early detection of exotic species. These methodologies are based on extracting DNA directly from environmental samples (water, sediments) and identify the species present there from DNA traces. The techniques are becoming cheaper, are non-invasive, highly sensitive, independent of weather conditions for sampling, and may help to control target species [35]. There are several examples of molluscs' detection using eDNA from European waters, both invasive species such as *Rangia cuneata* [36], *Dreissena polymorpha* [37], *Xenostrobus securis* [38], *Potamopyrgus antipodarum* [39] and locally endangered natives like *Margaritifera margaritifera* [40].

The main objectives of this study were two-fold. First to design an easy and fast method to detect presence-absence of four freshwater molluscs invasive to Europe from eDNA: *Corbicula fluminea*, *Melanoides tuberculata*, *Mytilopsis leucophaeata* and *Sinanodonta woodiana* based on simple PCR. Second, to validate the new primers in situ testing the hypothesis of positive eDNA results in the areas where either conventional sampling and/or official records were obtained. For this, samples upstream, within and downstream the three most populated non coastal Spanish cities were employed. Expectations were sampling points within and downstream cities provided more positives, given reported association between human population density and invasive species [28–31].

## Materials and methods

### Study region and target species

The mollusc species analysed in this study (*Corbicula fluminea*, *Melanoides tuberculata*, *Mytilopsis leucophaeata*, *Sinanodonta woodiana*) are considered invasive in the Iberian Peninsula. They are included in the current official list of invasive species in Spain (Spanish Directive of 4 August 2013 RD 630/2013).

Three main rivers near to the most populated cities inland the Iberian Peninsula were selected to evaluate the potential association of these invasive species with urban areas. Manzanares River crosses Madrid (3 165 000 inhabitants) and is a tributary of Tajo River (the longest river basin of the Iberian Peninsula). Guadalquivir River crosses Sevilla (690 000 inhabitants), and Ebro River crosses Zaragoza (661 000 inhabitants). Water samples were collected upstream (Point 1) (coordinates: Madrid 40.404968N, 3.722536W, Sevilla 37.404152N, 5.998669W and Zaragoza 41.736952N, 0.992233W), within (Point 2) (coordinates: Madrid 40.400108N, 3.718048W, Sevilla 37.404307N, 5.998946W and Zaragoza 41.658574N, 0.878066W) and downstream (Point 3) (coordinates: Madrid 40.326673N, 3.654334W, Sevilla 37.403653N, 6.006897W and Zaragoza 41.632217N, 0.837865W) each considered city. Two additional points were sampled from upstream enclosed areas with reported occurrence of one of these species: Santillana reservoir (River Manzanares coordinates 40.719003N, 3.855379W) and the lake of Alhama de Aragón (Jalón River, Ebro’s tributary, coordinates 41.294383N, 1.898593W), which have respectively *Sinanodonta woodiana* (Madrid Community Official Bulletin Decreto 102/2014 of 8 September 2014) and *Melanoides tuberculata* [17].

Sampling included: taking water samples (see "Water sample collection and eDNA extraction" section below), and *a posteriori* intensive search of the invasive species found in a site from water eDNA, if any. For comparable sampling effort, the bottom (stones, pebbles, sediments) were carefully inspected from an area of 5m^2^ in each revisited sampling point until the species was found, or during one hour.

No specific permissions were required for sampling in these locations; all of them are of public access and the species: *C*. *fluminea*, *M*. *leucophaeata*, *M*. *tuberculata* and *S*. *woodiana* are not native from Spain.

### Specific primers design

The design and validation of taxon-specific primers was based on the methodology described by Clusa *et al*. [39] and Ardura *et al*. [37]. 16S rRNA and COI (cytochrome oxidase subunit I) genes were selected to design the specific marker, since they are mitochondrial and expected to be in abundance in eDNA [41]. All the 16S and COI sequences from the target species, related species and other aquatic species of a wide range of taxa were downloaded from the NCBI database. All different haplotypes were visualized with BioEdit [42] and the sequences were aligned with the ClustalW application included therein [43]. A region conserved in all the haplotypes of the target species but different in the rest of species was searched and used to design two specific primers per species. Both primers were tested with the Oligo Analyzer 3.1 tool included in the Integrated DNA Technologies webpage (http://eu.idtdna.com/calc/analyzer) in order to obtain similar annealing temperature and to check the primers not to form hairpins or dimers.

### Primers validation

The first validation step was to check the primers *in silico* by an alignment with the BLAST tool of the NCBI webpage [44]. For *in vitro* validation, tissue from muscle from different molluscs and fishes from the laboratory were used to check for possible cross-species amplification of the designed primers. The four genera are absent in the Iberian Peninsula [45, 46]. Therefore species that belong to different families including molluscs which are common in the Iberian Peninsula (S1 Table) were selected for the *in vitro* assays. To discard the not very likely but possible cross-reactivity with fish species we included four fishes from different families that are abundant in Spanish waters (S1 Table). DNA was extracted from tissue with Chelex resine [47] in the case of fish samples and with the mollusc DNA Extraction Kit (Omega Bio-Tek, USA) following the instructions provided by the manufacturer in the case of mollusc samples. To confirm good DNA quality in each sample COI gene was amplified with universal primers following the protocol described by Geller *et al*. [48]. Thus absence of PCR amplification with specific primers cannot be attributed to lack of good DNA in a sample but to the absence of the target species. All the markers were tested on all the eDNA samples. The sensitivity of the specific primers was determined *in vitro* with serial dilutions of the target species DNA from a known concentration quantified with Qubit 2.0 fluorimeter (Invitrogen, ThermoFisher Scientific) following the same protocol described by Clusa *et al*. [39]. Stocks concentrations were in the range of 1–5 μg/ml. Dilutions made were: 1:5; 1:10; 1:25; 1:50; 1:100; 1:500; 1:1000; 1:5000; 1:10000; 1:50000 and 1:100000. The previous concentration to the one where no amplification was observed in agarose gel was considered the detection limit.

### Water sample collection and eDNA extraction

From January to April 2016, two replicates of 1L water were collected with sterile bottles from each sampling point, putting the bottle as close to the bottom substrate as possible. The different sites were sampled in different days and always from upstream to downstream. All the material was cleaned with bleach between samplings following Goldberg *et al*. [49], to ensure decontamination of the equipment. Samples were immediately frozen and transported to the laboratory. Water samples were vacuum filtered using the Supor®-200 Membrane Filter (Pall Corporation) with 0.2 μm pore size and a filter holder. The filter holder was dismantled, sprayed with 10% bleach, cleaned with detergent and 10% bleach, rinsed with distilled water and sterilized by 30 minutes under UV light between samples. Filters were stored individually within 15ml tubes at -20°C until DNA extraction. DNA was extracted with the PowerWater® DNA Isolation Kit (Mobio laboratories) following manufacturer’s recommendations. The two replicates of each sampling point were extracted separately in time. The eDNA extractions were done under sterile conditions, in a laboratory unit where there were no other tissue samples, inside a PCR laminar flow cabinet treated with ultraviolet light to avoid any contamination of the environmental DNA. To ensure the cleaning process was correct, one sample with 1L milliQ water was filtrated between two problem samples and included in all eDNA analyses to confirm that contamination did not occur during the filtration or DNA extraction process.

### PCR conditions

The amplification reaction with the species-specific primers from tissue DNA was performed in a total volume of 20μl, including Green GoTaq® Buffer 1X, MgCl2, 0.25mM dNTPS, 1μM of each primer, 2μl of template DNA and 0.65 U of DNA Taq polymerase (Promega). The PCR conditions were the following: an initial denaturation step at 95ºC for 5min, 35 cycles at 94ºC for 30s, annealing at the temperature of choice for 30s and elongation at 72ºC for 30s. A final step of elongation was set at 72ºC for 10min. We assayed different annealing temperatures and MgCl_2_ concentrations for each pair of primers. In every PCR a positive control using tissue DNA of the target species (from voucher specimens kindly provided by the Museo Nacional de Ciencias Naturales, Madrid) and a negative control containing PCR reagents and distilled water (to discard contamination during preparation of PCR) were included. PCR products were visualized in 2% agarose gels with 2.5μl of SimplySafe™.

The individuals sampled in situ were Barcoded for the COI gene using Geller *et al*. [48] primers jgLCO1490 and jgHCO2198, for species confirmation, with the PCR conditions indicated by the authors and using muscle tissue as DNA source. DNA extraction from tissue was as reported above. The new designed primers were used to amplify their DNA as well.

In the case of DNA extracted from water samples, the PCR conditions were the same as described above with some minor modifications. Fifty cycles were used instead of 35; 6μl of DNA template and BSA (200ng/ml) was added in the PCR mix. In addition to the positive and negative controls for PCR, a negative control for extraction was included. All the PCRs from eDNA were done in a PCR cabinet where no tissue sample was handled, treated with ultraviolet light before preparing the mix in order to avoid contamination of the samples and using pipette filter tips. The positive control was added outside the cabinet and separately from water samples.

All the positive bands obtained from eDNA were purified following instructions either with the Exo-BAP (EURx) or cutting the band with the agarose out DNA purification kit (EURx) in case of multiple bands and sequenced to confirm the species. In each eDNA replica two PCRs were done for each species. A minimum of two positive amplifications from one extraction or one positive result from two different extractions were required to consider a species was present in a sample.

### Ethics statement

DNA from fish species were from the laboratory collection. The study was approved by the Ethics Committee from the Principality of Asturias with the permit of reference number 99/16 (for the project MINECO-13-CGL2013-42415-R) and with the permit of reference number 101/16 (for the project EU RIA 689682 –AMBER).

## Results

### Specific markers

The specific primers designed for the target species are shown in Table 1. From the BLAST test, the combination of the two new primers for each species retrieved significant alignments only with the target species, except in the case of *Corbicula fluminea*. For this last species it was not possible to find a specific marker nor in 16S or COI. A *Corbicula* genus-specific marker was designed within the 16SrDNA gene that anneals, from BLAST, on *C*. *leana* (a synonym of *C*. *fluminea* [50]) and *C*. *largillierti*, which is native to Asia and invasive in South America [51, 52].

**Table 1:** Taxon-specific primers designed in this study.

Species detected	Primer	Sequence (5’-3’)	Annealing Temperature	[Mg^2+^]	Amplicon size	Detection limit
*Corbicula sp*	CoFl-16S-F	GAATAACTTAAATGTAGGT	55°C	2 mM	165 bp	0.375 ng/ml
	CoFl-16S-R	AGCAAACTTCTTCTTAAATAT				
*Melanoides tuberculata*	MeTu-16S-F	GGTCTRACGAAAGCAATACT	58°C	2 mM	230 bp	3 ng/ml
	MeTu-16S-R	GCTTTGCTKGATCTAAAYYT				
*Mytilopsis leucophaeata*	MyLe-COI-F	GGTTGTAACAACGCACGGTTTAG	66°C	1 mM	193 bp	0.76 ng/ml
	MyLe-COI-R	CACCTTCTCTGAAAGCCGAGC				
*Sinanodonta woodiana*	SiWo-COI-F	GGGTCAGCCMGGRAGGCTTTTA	68°C	1 mM	258 bp	0.202 ng/ml
	SiWo-COI-R	TGTTCACCCTGTACCAACRCCC				

Primer’s sequence, annealing temperature, Mg^2^ concentration, expected amplicon size (in base pairs) and minimum DNA concentration (detection limit) for which is possible to obtain a PCR product visible in agarose gel with the primer pairs in the conditions assayed.

**Fig 1:**
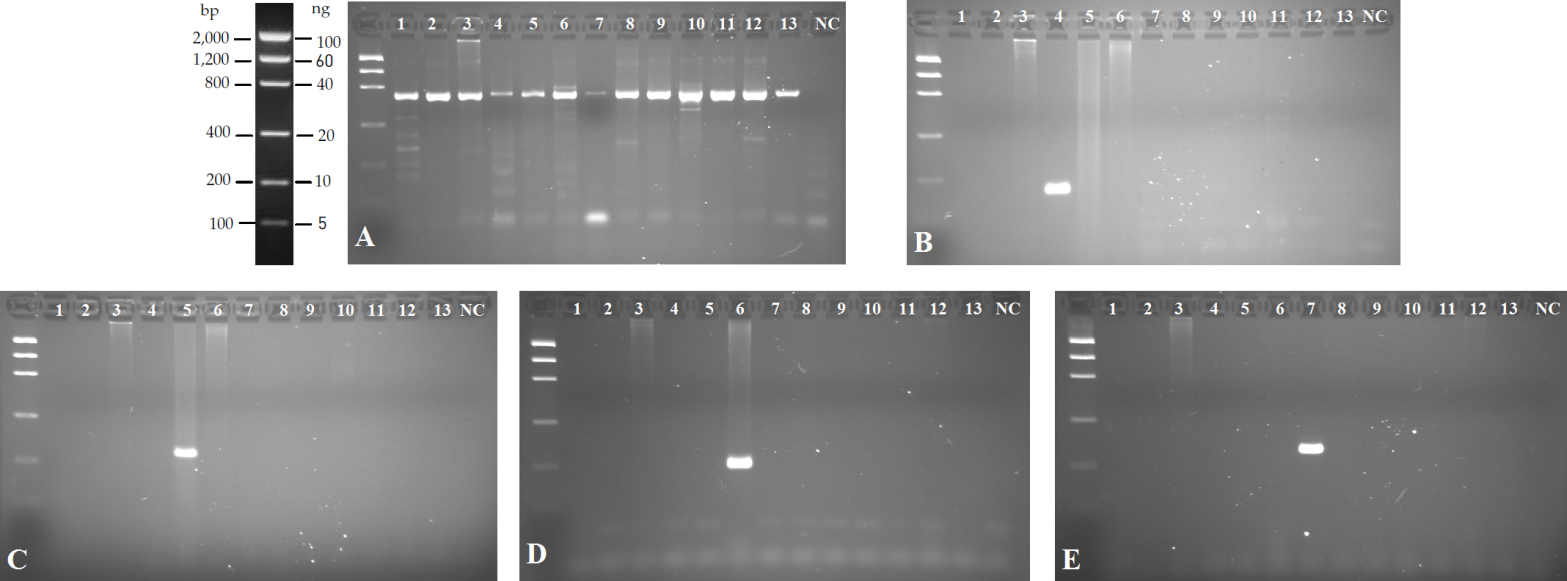
Agarose gels (2%) showing the results of cross-amplification experiments for each specific marker. PCR amplified with: A) universal primers [48]; and specific primers for *Corbicula sp*. (B), *Melanoides tuberculata* (C), *Mytilopsis leucophaeata* (D); *Sinanodonta woodiana* (E). Lanes (from 1 to 13) in all gels are: Ladder, 1-*Mya arenaria*, 2-*Rangia cuneata*, 3-*Dreissena polymorpha*, 4-*Corbicula fluminea*, 5-*Melanoides tuberculata*, 6-*Mytilopsis leucophaeata*, 7-*Sinanodonta woodiana*, 8-*Potamopyrgus antipodarum*, 9- *Bithynia tentaculata*, 10-*Salmo trutta*, 11-*Phoxinus phoxinus*, 12- *Carassius auratus*, 13-*Micropterus salmoides*, NC- Negative control.

In the cross-amplification test, the set of primers were tested against the collection of molluscs and fish species described above (S1 Table). All samples employed provided positive amplification with universal primers [48], confirming the presence of good quality DNA (Fig 1A). For the newly designed taxon-specific primers no amplification was found from any of the aquatic species assayed except from DNA of the target species of each primer (Fig 1B, 1C, 1D and 1E). The detection limit for each marker (Table 1) was close to 1 ng/ml for all the species.

### Field results

**Fig 2:**
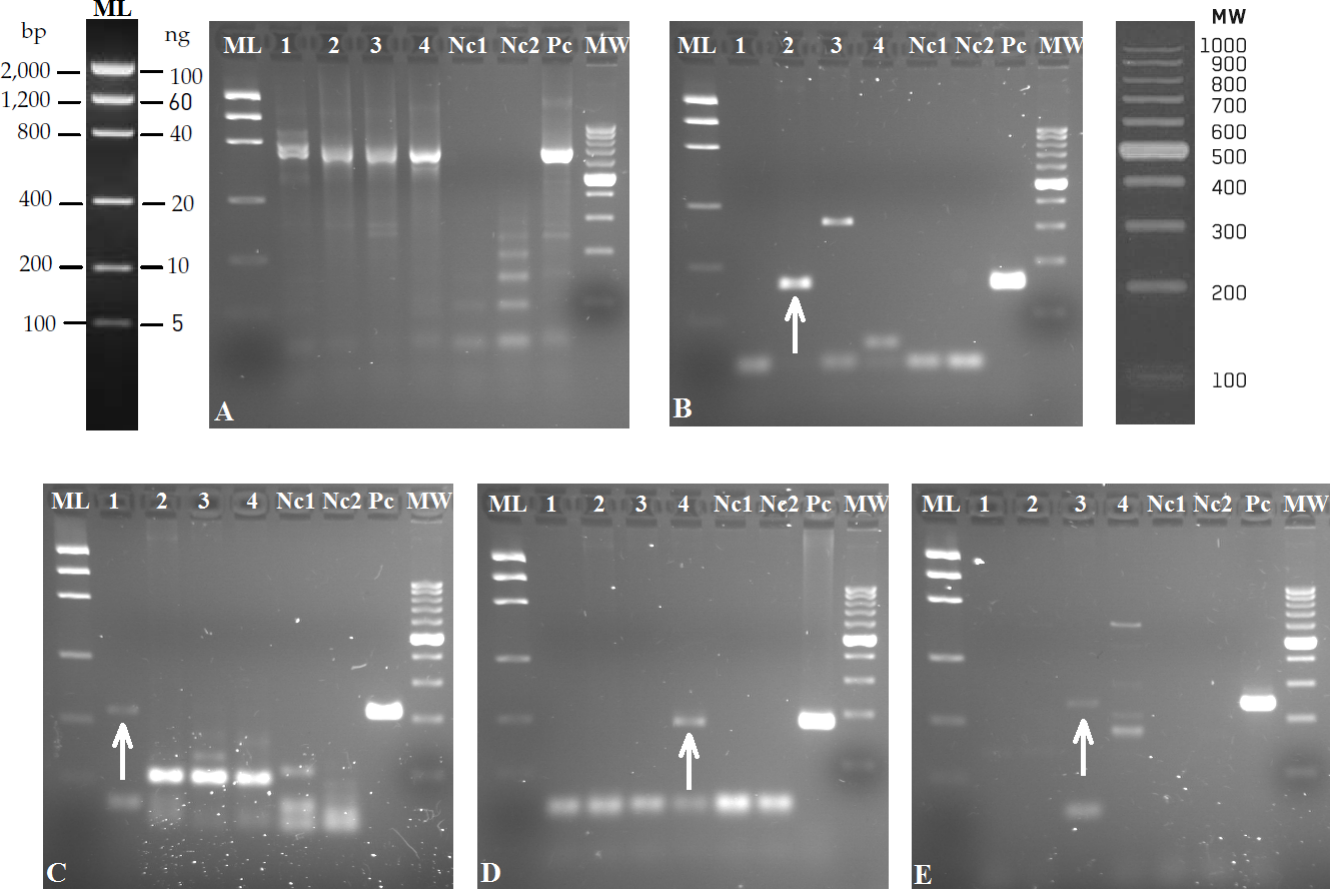
Agarose gels (2%) showing amplification products obtained from eDNA with the designed specific markers. PCR amplicons with: A) universal primers [48]; and specific primers for *Corbicula sp*. (B), *Melanoides tuberculata* (C), *Mytilopsis leucophaeata* (D); *Sinanodonta woodiana* (E). Lanes in all gels are: Mass ladder, 1-Alhama de Aragón thermal lake, 2-Ebro River (Zaragoza), 3-Santillana reservoir (Madrid), 4-Guadalquivir River (Sevilla), Nc1- Negative control for extraction, Nc2 negative control for PCR and Pc positive control with tissue DNA of each species.

The four designed markers provided positive amplification from real eDNA samples (Fig 2), in the three rivers. All eDNA samples were positively PCR-amplified with universal primers [48], thus eDNA was of sufficient quality for PCR amplification and PCR inhibitors did not occur (Fig 2A). *Corbicula* specific primers amplified from the three Ebro River samples (Fig 2B, Table 2); one individual was sampled in point 2 in the city centre (GenBank accession number MF401395). *M*. *tuberculata* specific primers amplified from Alhama de Aragón lake only (Fig 2C), where several individuals were collected (GenBank accession number MF401394). *M*. *leucophaeata* primers provided positive PCR amplification in two of the three samples from Guadalquivir River (near Sevilla) (Fig 2D, Table 2), where several individuals were collected (GenBank accession number MF401396). *S*. *woodiana* primers amplified DNA fragments from Santillana reservoir sample (Fig 2E). Negative control for extraction (1L of milliQ water filtrated and extracted at the same time as the rest of eDNA samples, NC1 in Fig 2; Table 2) was clean for every marker, indicating the absence of contamination during the filtration and eDNA extraction process. Also negative control for PCR (distilled water added to the PCR mix instead of DNA, NC2 in Fig 2) was clean, indicating the absence of contamination while preparing the PCR. There are some unspecific bands in the eDNA analysis, but not of the same size of the target species (Fig 2). The bands marked with an arrow on Fig 2 were excised from the gel, the amplicons sequenced, and the species was confirmed by BLAST (S2 Table) (DDBJ accession numbers LC310741-LC310751). From BLAST tests, the species amplified from Ebro River samples was *Corbicula fluminea*. Regarding urban river areas, only the river zone around Madrid did not provide positive PCR amplification for any of the assayed markers; in the two other urban areas positive amplification was found for one of the assayed species (Table 2).

**Table 2:** PCR amplification using the new taxon-specific primers on eDNA obtained from water samples of the considered Iberian rivers.

	Manzanares River basin				Guadalquivir River basin			Ebro River basin				
	Santillana reservoir MR	Madrid M1	Madrid M2	Madrid M3	Sevilla S1	Sevilla S2	Sevilla S3	Jalón River lake EL	Zaragoza Z1	Zaragoza Z2	Zaragoza Z3	
Coordinates	40.719003N, 3.855379W	40.404968N, 3.722536W	40.400108N, 3.718048W	40.326673N, 3.654334W	37.404152N, 5.998669W	37.404307N, 5.998946W	37.403653N, 6.006897W	41.294383N, 1.898593W	41.736952N, 0.992233W	41.658574N, 0.878066W	41.632217N, 0.837865W	Extraction negative control
*Corbicula sp*	-	-	-	-	-	-	-	-	X	X	X	-
*Melanoides tuberculata*	-	-	-	-	-	-	-	X	-	-	-	-
*Mytilopsis leucophaeata*	-	-	-	-	X	X	-	-	-	-	-	-
*Sinanodonta woodiana*	X	-	-	-	-	-	-	-	-	-	-	-
COI [48]	X	X	X	X	X	X	X	X	X	X	X	-

Positive PCR amplification is shown with X. Negative PCR is indicated as "-".

## Discussion

In this study we have developed a useful PCR-based tool to detect four of the main freshwater molluscs invasive to Europe from water samples. Positive amplification of the expected species from water samples was found for the places where the species had been sampled, such as *Melanoides tuberculata* in Alhama de Aragón lake [17] and *Sinanodonta woodiana* in Santillana reservoir within Manzanares River (Madrid Community Official Bulletin Decreto 102/2014 of 8 September 2014). Although the primers were tested only in Spanish waters they are species- or genus-specific and did not show any cross amplification with other species either in the BLAST assays or in the *in vitro* tests. Thus they could be potentially useful to monitor these molluscs across Europe and other regions where they are invasive, as well as in native areas as proposed for markers developed for other species [39]. Unspecific bands were observed in the eDNA analysis, but not of the same size, similar to other studies [38, 39]. For that reason positive control in the PCR is highly recommended, first to correctly identify the band size of the target species and secondly to discard false negatives due to failure in the PCR [49]. Additionally the sequencing of the possible positive could help to elucidate the species amplified [49]. In the case described here, all the eDNA PCR results were purified and sequenced identifying the species present in the samples as the target species confirming the correct detection of the invasive species.

Two of these species were found in urban river areas nearby two of the most populated Spanish cities. *Corbicula fluminea* DNA was found from all the sampling points tested from Ebro River. Oscoz *et al*. [17] indicated that the invasion of this species had not reached Zaragoza city in 2010, but in this study, carried out six years later, we found it in the city and 5.44 kilometres downstream (sampling points Z1-Z3) (Table 2), demonstrating the expansion of this invasive species. The rest of the rivers tested seem to be free of the *Corbicula* invasion but it is necessary to continue the surveillance and control the possible introduction of the species.

The other species associated with city areas was *Mytilopsis leucophaeata*, found in the section of Guadalquivir River crossing Sevilla (points S1 and S2). The species had been described in this river 14 years ago, restricted to an enclosed channel of water distribution for refrigeration [7]. Although this species is considered to have relatively reduced dispersal capacity [7, 53], its presence in open river waters would indicate the species started already spreading along the basin, at least in the urban zone sampled in this study.

The occurrence of *Melanoides tuberculata* in the Ebro River basin seems to be still restricted to the place where it had been already described; which is a lake with high water temperature as preferred by this species [54, 55]. In 2010 Jarillo and Salgado [56] reported its presence in L'Aldea in the Ebro's Delta, but the survival rate was too low. If climate change continues raising water temperatures, *M*. *tuberculata* would be a threat for the rest of the species in the region, as already happened in Alhama de Aragón lake where it is displacing the local native *Melanopsis penchinatti*, now classified as critically endangered [57].

On the other hand, *Sinanodonta woodiana* seems to be also restricted to an enclosed area (a reservoir) within Manzanares River and has not reached downstream running waters yet (or at least its DNA, if present, is at a very low concentration below the detection limit of 0.202 ng/ml found for this marker). Perhaps the reservoir dam represents a barrier to the expansion of this species, as it is for the migration of other species e.g. [58]. *S*. *woodiana* was not detected in Ebro River, although it is reported in Ter River and Daró River (north East Rivers) [59]. Numerous invasive species have been translocated from these Rivers to Ebro River and vice versa as an example *Misgurnus anguillicaudatus* [60]. In any case, since it may spread upstream the presence of the species in the basin is a potential threat for native molluscs as *Margaritifera auricularia* [61].

Finally, eDNA-based methodologies are not perfect for river faunal inventories and could be considered exploratory or early-detection systems instead. Floating DNA (not actual individuals) may be transported downstream creating false positives [62]. To deal with the possibility of false positives in the eDNA analysis an unambiguous detection approach was used as described in Lahoz-Monfort *et al*. [63]. Conventional sampling and/or fully referenced and reliable official reports confirmed the presence of the species in all the places where positive results were obtained for eDNA. *Corbicula fluminea* individuals were found in Ebro River Z2 sample, *Melanoides tuberculata* were found in Alhama de Aragón Lake, *Mytilopsis leucophaeata* were found in Sevilla samples and *Sinanodonta woodiana* has been reported in Santillana reservoir sample. Moreover replicates were considered in each place. Two samples from each sampling site were collected and extracted separately in time. In each eDNA sample two PCRs were done, thus a total of four replicates were used. Finally, all the positive results from eDNA were sequenced to confirm the species.

On the other hand, when a species is very scarce in a place and its DNA has very low concentration false negatives may occur, especially if the detection limit is not too low. In addition sampling design is also important when working with eDNA. The possible false negatives could derive from the life cycle of the target species, since many species vary their activity depending on season [64, 65]. eDNA will be more effective when sampling is done according to seasonal activity of the species. Ardura *et al*. [36, 37] collected eDNA samples during spawning session for two invasive molluscs, *Rangia cuneata* and *Dreissena polymorpha* respectively, in order to use specific markers to detect them. The use of replicates, both temporal and spatial (from the same sampling point) is highly recommendable [66]. In any case, detecting eDNA of an- unreported potentially invasive species in a location should be followed by intensive conventional sampling to corroborate the invasion status. The new markers developed here could serve for the early detection step. Although the method described is non-quantitative, since it only determines the presence-absence of the invasive species, it is cheaper and faster than qPCR [67] and as reliable as qPCR or ddPCR to inference species presence in a sample [68]. It could be adapted to be used with qPCR but as it is it would be useful to help managers to control the spread of these invasive species, especially in places where only presence data is required or with limited resources.

## Conclusion

Four eDNA-based markers were successfully designed, validated and applied in situ in Iberian rivers for detecting DNA from the highly invasive molluscs *Corbicula spp*, *M*. *tuberculata*, *M*. *leucophaeata and S*. *woodiana*. The new tools are ready to be used in other regions where these species are also invasive and could help to control their spreading.

## Supporting information

S1 Table Taxonomy of the species used in the cross amplification test. Different molluscs and fishes from a variety of taxa were chosen.
(DOCX)

S2 Table Sequences obtained with the new specific primers from positive control (tissue DNA) and environmental samples (eDNA). (DOCX)
