# Supplementary material for: eDNA for detection of five highly invasive molluscs. A case study in urban rivers from the Iberian Peninsula
Source: PLoS One. 2017 Nov 15;12(11):e0188126. doi: 10.1371/journal.pone.0188126 (PMC5687721; doi:10.1371/journal.pone.0188126)
Supplement: S1 Table — Different molluscs and fishes from a variety of taxa were chosen. (DOCX) [file pone.0188126.s001.docx]

**S1 Table. Taxonomy of the species used in the cross amplification test.** Different molluscs and fishes from a variety of taxa were chosen.

| **Kingdom** | **Phylum** | **Class** | **Order** | **Family** | **Genus** | **Species** |
| --- | --- | --- | --- | --- | --- | --- |
| Animalia | Mollusca | Bivalvia | Myida | Dreissenidae | *Dreissena* | *Dreissena polymorpha* |
| Animalia | Mollusca | Bivalvia | Myida | Dreissenidae | *Mytilopsis* | *Mytilopsis leucophaeata* |
| Animalia | Mollusca | Bivalvia | Myida | Myidae | *Mya* | *Mya arenaria* |
| Animalia | Mollusca | Bivalvia | Unionoida | Unionidae | *Sinanodonta* | *Sinanodonta woodiana* |
| Animalia | Mollusca | Bivalvia | Veneroida | Corbiculidae | *Corbicula* | *Corbicula fluminea* |
| Animalia | Mollusca | Bivalvia | Veneroida | Mactridae | *Rangia* | *Rangia cuneata* |
| Animalia | Mollusca | Gastropoda | Sorbeoconcha | Thiaridae | *Melanoides* | *Melanoides tuberculata* |
| Animalia | Mollusca | Gastropoda | Littorinimorpha | Bithyniidae | *Bithynia* | *Bithynia tentaculata* |
| Animalia | Mollusca | Gastropoda | Littorinimorpha | Tateidae | *Potamopyrgus* | *Potamopyrgus antipodarum* |
| Animalia | Chordata | Actinopterygii | Cypriniformes | Cyprinidae | *Phoxinus* | *Phoxinus phoxinus* |
| Animalia | Chordata | Actinopterygii | Cypriniformes | Cyprinidae | *Carassius* | *Carassius auratus* |
| Animalia | Chordata | Actinopterygii | Perciformes | Centrarchidae | *Micropterus* | *Micropterus salmoides* |
| Animalia | Chordata | Actinopterygii | Salmoniformes | Salmonidae | *Salmo* | *Salmo trutta* |
