# Supplementary material for: eDNA for detection of five highly invasive molluscs. A case study in urban rivers from the Iberian Peninsula
Source: PLoS One. 2017 Nov 15;12(11):e0188126. doi: 10.1371/journal.pone.0188126 (PMC5687721; doi:10.1371/journal.pone.0188126)
Supplement: S2 Table — (DOCX) [file pone.0188126.s002.docx]

**S2 Table. Sequences obtained with the new specific primers from positive control (tissue DNA) and environmental samples (eDNA)**.

| **Sample** | **Species** | **Source** | **forward primer name** | **forward primer sequence (5'-3')** | **reverse primer name** | **reverse primer sequence (5'-3')** | **SEQUENCE (5'-3')** |
| --- | --- | --- | --- | --- | --- | --- | --- |
| CoFl-sp | *Corbicula fluminea* | Tissue –individual Z2 point | CoFl-16S-F | GAATAACTTAAATGTAGGT | CoFl-16S-R | AGCAAACTTCTTCTTAAATAT | Cf-E2: ATTGGGGCAATAGAAAATGAAATGAATCATTTTTTTTATTATAAGGATCCAGTTTTGACTGAAAAAAGCAAAAGCTACCGCGGGGATAACAGGGTAATTTTTTCTGAGAGTTCATATTTAAGAAGAAGTTTGCT |
| Ebro1_CF | *Corbicula fluminea* | Ebro River eDNA Z1 point | CoFl-16S-F | GAATAACTTAAATGTAGGT | CoFl-16S-R | AGCAAACTTCTTCTTAAATAT | Cf-eE1:  GAAATGAATCATTTTTTTTATTATAAGGATCCAGTTTTGACTGAAAAAAGCAAAAGCTACCGCGGGGATAACAGGGTAATTTTTTCTGAGAGTTCATATTTAAGAAG |
| Ebro2_CF | *Corbicula fluminea* | Ebro River eDNA Z2 point | CoFl-16S-F | GAATAACTTAAATGTAGGT | CoFl-16S-R | AGCAAACTTCTTCTTAAATAT | Cf-eE2:  AAATGAAATGAATCATTTTTTTTATTATAAGGATCCAGTTTTGACTGAAAAAAGCAAAAGCTACCGCGGGGATAACAGGGTAATTTTTTCTCGTAGAG |
| Ebro3_CF | *Corbicula fluminea* | Ebro River eDNA Z3 point | CoFl-16S-F | GAATAACTTAAATGTAGGT | CoFl-16S-R | AGCAAACTTCTTCTTAAATAT | Cf-eE3: AATGAATCATTTTTTTTATTATAAGGATCCAGTTTTGACTGAAAAAAGCAAAAGCTACCGCGGGGATAACAGGGTAATTTTTTCTGAGAGTTCATATT |
| MeTu-sp | *Melanoides tuberculata* | Tissue –individual EL point | MeTu-16S-F | GGTCTRACGAAAGCAATACT | MeTu-16S-R | GCTTTGCTKGATCTAAAYYT | Mt-EL: GTAGGTGAAGAGGCCTATATTATATTGAAGGACAAGAAGACCCTGTCGAGCTTTAAAATTAAATATAGGTGTAAATTATTAACAAAATCAATGAACTGCATTATATTTTTAGTTGGGGCGACGAAGGAACAAACAAAGCTTCTTTTATTTTTATAAATTTATAGGTTTAGATCCAGCAAAGC |
| Alh-MT | *Melanoides tuberculata* | Lake eDNA EL point | MeTu-16S-F | GGTCTRACGAAAGCAATACT | MeTu-16S-R | GCTTTGCTKGATCTAAAYYT | Mt-eEL:  TATTGAAGGACAAGAAGACCCTGTCGAGCTTTTAAATTAAAGTAGGTGTAAATTATTAACAAAATCAATGAACTGCATTACATTTTTAGTTGGGGCGACGAAGGAACAAACAAAGCTTCTTTTATTTTTATAAATTTATAGGTTTAGATCCAGCAAAGC |
| MyLe-sp | *Mytilopsis leucophaeata* | Tissue – individual S1 point | MyLe-COI-F | GGTTGTAACAACGCACGGTTTAG | MyLe-COI-R | CACCTTCTCTGAAAGCCGAGC | Ml-G1: CCTATAATGATGGGTGGTTTTGGAAATTGATTAGTTCCAATAATACTAGCAGTGCCTGATATAGGATTTCCTCGTTTAAATAATGTTAGGTTTTGGGTGTTACCTGTATCTATAGGTCTTTTATTTTGCTCGGCTTTCAGAGAAGGTG |
| Sevilla1_ML | *Mytilopsis leucophaeata* | Guadalquivir River eDNA S1 point | MyLe-COI-F | GGTTGTAACAACGCACGGTTTAG | MyLe-COI-R | CACCTTCTCTGAAAGCCGAGC | Ml-eG1:  TGGGTGGTTTTGGAAATTGATTAGTTCCAATAATACTAGCAGTGCCTGATATAGGATTTCCTCGTTTAAATAATGTTAGGTTTTGGGTGTTACCTGTATCTATAGGTCTTTTATTTTGCTCGGCTTTCAGAGAAGGTG |
| Sevilla2_ML | *Mytilopsis leucophaeata* | Guadalquivir River eDNA S2 point | MyLe-COI-F | GGTTGTAACAACGCACGGTTTAG | MyLe-COI-R | CACCTTCTCTGAAAGCCGAGC | Ml-eG2: GATGGGTGGTTTTGGAAATTGATTAGTTCCAATAATACTAGCAGTGCCTGATATAGGATTTCCTCGTTTAAATAATGTTAGGTTTTGGGTGTTACCTGTATCTATAGGTCTTTTATTTTGCTCGGCTTTCAGAGAAGGTG |
| SiWo-sp | *Sinanodonta woodiana* | Tissue – museum specimen | SiWo-COI-F | GGGTCAGCCMGGRAGGCTTTTA | SiWo-COI-R | TGTTCACCCTGTACCAACRCCC | Sw-voucher:  TGTTACGGCTCATGCTTTTATAATAATTTTCTTCTTAGTTATACCTATAATGATTGGAGGGTTTGGGAATTGATTAATTCCTTTAATAATTGGGGCTCCTGATATGGCTTTTCCTCGATTGAATAATTTAAGGTTTTGGTTACTTGTGCCAGCGCTATTTTTATTATTAAGGTCTTCTTTGGTGGAAAGGGGCGTTGGTACAGGGTGAACA |
| Sant_SW | *Sinanodonta woodiana* | Reservoir eDNA MR point | SiWo-COI-F | GGGTCAGCCMGGRAGGCTTTTA | SiWo-COI-R | TGTTCACCCTGTACCAACRCCC | Sw-eMR: ATAATTTTCTTCTTAGTTATACCTATAATGATTGGAGGGTTTGGGAATTGATTAATTCCTTTAATAATTGGGGCTCCTGATATGGCTTTTCCTCGATTGAATAATTTAAGGTTTTGGTTACTTGTGCCAGCGCTATTTTTATTATTAAGGTCTTCTTTGGTGGAAAGGGGCGTTGGTACAGGGTGAACA |
